# Occurrence of *Babesia* Species and Co-Infection with *Hepatozoon canis* in Symptomatic Dogs and in Their Ticks in Eastern Romania

**DOI:** 10.3390/pathogens10101339

**Published:** 2021-10-17

**Authors:** Lavinia Ciuca, Gabriela Martinescu, Liviu Dan Miron, Constantin Roman, Dumitru Acatrinei, Giuseppe Cringoli, Laura Rinaldi, Maria Paola Maurelli

**Affiliations:** 1 Department of Veterinary Medicine and Animal Production, University of Naples Federico II, CREMOPAR Via Delpino, 1, 80137 Naples, Italy; cringoli@unina.it (G.C.); laura.rinaldi@unina.it (L.R.); mariapaola.maurelli@unina.it (M.P.M.); 2 Parasitology Service, Clinics Department, Faculty of Veterinary Medicine, “Ion Ionescu de la Brad” Iasi University of Life Sciences (IULS), Mihail Sadoveau Alley, 3, 700489 Iasi, Romania; lmiron@uaiasi.ro (L.D.M.); roman.const@yahoo.com (C.R.); dacatrinei@yahoo.com (D.A.)

**Keywords:** *Babesia* species, *B. rossi*, dogs, ticks, Romania

## Abstract

Although the distribution of *Babesia* spp. and *Hepatozoon canis* is well known in Romania, there is still a marked lack of information in many places of the country. This study aimed to investigate the occurrence of these haemoparasites in symptomatic dogs and in their ticks in Iasi, eastern Romania. Ninety owned dogs were subjected to clinical examination at the Faculty of Veterinary Medicine of Iasi and all detectable ticks (58 ticks from 15 dogs) were collected. Additionally, 124 ticks collected from the coat of other dogs (no. = 23) were included. Three *Babesia* species were found in dogs: *Babesia canis* (94.4%), *Babesia vogeli* (3.3%), and *Babesia rossi* (2.2%). All the dogs resulted negative for *H. canis*. The ticks were identified as follows: *Ixodes ricinus* (64%), *Dermacentor reticulatus* (33%), and *Rhipicephalus sanguineus* group (3%). *B. canis* (Minimum Infection Rate; MIR = 81%), *B. vogeli* (MIR = 3%), and *Babesia microti*-like piroplasm (MIR = 1%) were found in ticks. Moreover, 15 ticks were positive for *H. canis*, 6 were co-infected with *B. canis,* and 1 with *B. microti*-like piroplasm. This is the first molecular identification of *B. rossi* in two symptomatic dogs from Romania, although further studies are needed to investigate the vector competence of other ticks from Europe.

## 1. Introduction

In the last years, tick-borne pathogens (TBP) increased their prevalence and distribution, due to climate change, globalization, population movements, and growth, thus representing a serious problem for animals and humans [1,2,3]. Intracellular apicomplexan haemoparasites such as *Babesia* spp. and *Hepatozoon canis* are of particular concern to veterinarians because of the severe infections they cause in dogs and their wide geographic distribution [4,5,6,7,8,9,10,11]. Canine babesiosis, caused by different *Babesia* species, is transmitted by the bite of ixodid ticks, such as *Dermacentor reticulatus* (*Babesia canis*), *Rhipicephalus sanguineus* (*Babesia* *vogeli*), *Haemaphysalis leachi* and *Haemaphysalis* *elliptica* (*Babesia* *rossi*), *Haemaphysalis* *longicornis*, *Haemaphysalis* *bispinosa* and *R. sanguineus* (*Babesia* *gibsoni*), and involves erythrocytes [12,13,14,15]. Instead, *H. canis* is transmitted by ingestion of infected *R. sanguineus* group ticks and infects leukocytes [16,17]. The sexual reproduction for both *Babesia* spp. and *H. canis* occurs in the ticks, while asexual reproduction takes place in the vertebrae of intermediate hosts [2,18].

Clinical babesiosis are usually associated with anaemia and haemolysis, fever, and lethargy, and may progress to multi-organ failure with a risk of mortality [9,19,20]. The broad spectrum of clinical signs depends on the different degrees of virulence of the *Babesia* species, but also on several factors related to hosts including age, splenectomy, immune competence, and concomitant infections or diseases [21,22,23,24]. In addition, the severity of illness has been associated with high parasitaemia in *B. rossi* infections [25,26]. Other species, i.e., *B. vogeli* can cause a subclinical to moderate clinical disease with possibly severe haemolytic anaemia in young dogs [22]. Moreover, a new piroplasmid species, *Babesia negevi* n. sp., has been recently described in a dog with a fatal disease [27]. However, little is known regarding the correlation between the disease severity and parasite density in other *Babesia* species.

The infection caused by *H. canis* is usually asymptomatic in dogs and is associated with low parasitaemia and mild disease, but severe disease with fever, lethargy, cachexia, and anaemia can be associated with high level of parasitaemia [28,29,30].

In Romania, several studies provided data on the identification of *B. canis*, *B. vogeli,* and *B. gibsoni* in dogs by means of molecular and serological analyses [5,9,10,31,32]. Moreover, *H. canis* infection has been identified in dogs, foxes, and jackals in Romania [10,18,33,34]. Although the distribution of *Babesia* spp. and *H. canis* is well known in several parts of Romania, there is still a lack of information in many places of the country. Therefore, the aim of this study was to investigate the occurrence of *Babesia* species and the co-infection with *H. canis* in dogs suspected of babesiosis and in their ticks in Iasi, eastern Romania.

## 2. Results

### 2.1. Dogs

Thirty-five dogs (38.9%; 95% CI = 29.0–49.8) showed mild clinical signs and 55 (61.1%; 95% CI = 50.2–71.0) expressed the acute form, attributable to babesiosis. All 90 of the sampled dogs that tested positive for the babesial parasites using a stained thin blood smear were positive for the general piroplasmid PCR. Three *Babesia* species were found in dogs: *B. canis* (85/90 = 94.4%; 95% CI = 86.9–97.9) (99.1–100% identity with GenBank Accession numbers: MK571831), *B. vogeli* (3/90 = 3.3%; 95% CI = 0.9–10.1) (100% identity with GenBank Accession number: KY290979), and *B. rossi* (2/90 = 2.2%; 95% CI = 0.4–8.6) (100% identity with GenBank Accession Number: MT740273). All the dogs resulted negative for *H. canis.*

Additionally, the dogs that expressed acute clinical signs were positive for *B. canis* with the prevalence of 58.9% (95% CI = 48.0–69.0) and *B. rossi* with the prevalence of 2.2% (95% CI = 0.4–8.6). The dogs that showed mild clinical signs were positive for *B. canis* with the prevalence of 35.6% (95% CI = 25.9–46.4) and *B. vogeli* with the prevalence of 3.3% (95% CI = 0.9–10.1).

Prevalence values were higher in dogs between 1 and 3 years (58.9%; 95% CI = 48.0–69.0) and in males (52.2%; 95% CI = 41.5–62.7). The most frequently affected breeds were crossbreeds (52.2%; 95% CI = 41.5–62.8), followed by Peking dogs (14.4%; 95% CI = 8.2–23.8) and German Shepherds (10%; 95% CI = 4.9–18.6). All the dogs presented tick infestation histories and 74.4% (67/90; 95% CI = 63.9–82.8) had access to the outdoors. All the dogs received one or two doses of imidocarb-dipropionate (6.6 mg/kg, at two weeks apart; Imizol; Intervet) and additional supportive therapy, depending on the clinical disorders expressed. Moreover, five dogs have died (5.6%, four infected with *B. canis* and one with *B. rossi*) and for three of them the follow-up remains unknown. Overall, 91.1% of the dogs have recovered. The percentage of *B. canis*-infected dogs with access to the outdoors was significantly higher (*p* < 0.001) compared to dogs with an indoor lifestyle. Moreover, a statistically significant association was found between young dogs (1–3 years) and positivity for *B. canis* (*p* < 0.005). No statistically significant association was found between breed or gender and the positivity of *Babesia* species (Table 1).

### 2.2. Ticks

Dogs that presented with ticks during the routine visit were 33.6% of the total (38/113; 95% CI = 25.1–43.2). Briefly, 71.1% (27/38; 95% CI = 53.9–84.0) of dogs showed low infestation, 21.1% (8/38; 95% CI = 10.1–37.8) showed moderate infestation and 7.9% (3/38; 95% CI = 2.1–22.5) showed high infestation. A total of 182 ticks were collected from dogs; of these, 58 ticks were found on 15/90 (16.7%; 95% CI = 9.9–26.3) symptomatic dogs and 124 ticks on another 23 dogs previously analysed for piroplasmosis (as above described). Specifically, 179 adults (113 engorged females and 66 males), 2 nymphs, and 1 larva were found (Table 2). One hundred tick sub-samples were prepared and identified as follows: *Ixodes ricinus* (64%; 95% CI = 53.7–73.2), *Dermacentor reticulatus* (33%; 95% CI = 24.1–43.2), and the *Rhipicephalus sanguineus* group (3%; 95% CI = 0.8–9.1). *Babesia canis* (MIR = 81%; 95% CI = 71.7–87.9) (99.8–100% identity with GenBank Accession numbers: MK571831, accessed on 1 March 2021), *B. vogeli* (MIR = 3%; 95% CI = 0.78–9.6) (100% identity with GenBank Accession number: KY290979, accessed on 1 March 2021) and *B. microti*-like piroplasm (MIR = 1%; 95% CI = 0.1–6.2) (100% identity with GenBank Accession number: MN355504,) was found in ticks. In addition, 15 ticks were positive for *H. canis* (MIR = 15%; 95% CI = 8.9–23.9), six were co-infected with *B. canis*, and one with *B. microti*-like piroplasm (Table 2).

## 3. Discussion

We herein report the first comprehensive molecular survey of *Babesia* spp. and *H. canis* in owned dogs and in their ticks from the eastern part of Romania. More than 90% of the dogs and over 80% of the ticks collected from symptomatic dogs were positive for *B. canis*. Indeed, *B. canis* is a major cause of babesiosis in dogs from Romania, based on serological and molecular studies previously conducted in the southern and western parts of the country [5,6,8,35,36]. However, based on the findings reported by Andersson et al. [18], there might be a great variety of genotypes of this parasite in Romania [18]. Moreover, a high percentage of *B. canis* infected dogs with acute signs of clinical disease in Romania were imported from Hungary [5], thus highlighting that imported dogs with *B. canis* may play a critical role as reservoir hosts when introduced in areas free of this pathogen but with the presence of the vector tick.

More than 60% of the *B. canis*-infected dogs from the present study expressed acute clinical signs, which agrees with other studies conducted in Romania, Italy, and Hungary [5,9,37,38]. There are many reports attesting that the presence of *Babesia* species is highly associated with the geographical distribution of their vectors [5,39,40]. Several cases of canine babesiosis were reported from new areas of Germany, Hungary, Switzerland, and the Netherlands, due to the fact that *D. reticulatus* ticks were introduced in those areas [41,42,43,44,45]. However, *B. canis* has been found in areas where the presence of *D. reticulatus* is rare (e.g., the insular regions of Greece) [46]. The presence *of B. canis* and *B. vogeli* in dogs from the present study is also supported by the tick species that were found (*I. ricinus* = 64%, *D. reticulatus* = 33% and *R. sanguineus* group *=* 3%). In Romania, *D. reticulatus* was reported as the most common tick (67.2%) infecting dogs in the south, west, north, and central areas, followed by the *R. sanguineus* group (32.8%) [8,36,47,48]. The *Rhipicephalus sanguineus* group was also reported with an increased prevalence in the urban areas of Romania, including the southern area of Bucharest [49,50]. Only one study [51] has recently reported the presence of *D. reticulatus* in Iasi County, the same area from the present study, but the authors did not identify *I. ricinus* ticks as we reported herein. Moreover, almost half of all *I. ricinus* ticks removed from dogs were *Babesia*-positive, and the total prevalence of *B. canis* in *I. ricinus* ticks was higher than in *D. reticulatus* ticks (55% vs. 33%). However, additional studies are warranted to obtain further information of the actual spread of the tick species and their transmitted pathogens to dogs in the eastern part of Romania. We found no cases of *H. canis* infection in dogs, but 15 *I. ricinus* ticks were positive for *H. canis*, 6 were co-infected with *B. canis*, and 1 with *B. microti*-like piroplasm. The results are not in agreement with several studies from southern, western, central, and northern Romania, where we found an important prevalence of *H. canis* in both dogs and ticks, ranging from 15% to 48% [10,18,34]. However, our results showed that *H. canis* DNA was detected in *I. ricinus* ticks, similarly to the study by Andersson et al. [8]. *Hepatozoon* *canis* had been identified in *I. ricinus* ticks [52,53,54] collected from foxes and dogs, but this tick species is not acknowledged as a competent vector for *H. canis* [55]. Instead, there are other studies [18,56,57] which show that *H. canis* DNA was not detected in *R. sanguineus* south-eastern lineage ticks, or that report *H. canis* infections in areas where *R. sanguineus* ticks are not present [58], although this tick species is considered to be the main vector for *H. canis* [59,60]. On the other side, there are several studies that revealed the presence of co-infections with multiple tick-borne pathogens (TBP) in dogs, such as: *H. canis*, *Babesia* spp, *Ehrlichia canis,* and *Anaplasma platys* [61,62], as well as ticks that harbour the DNA of some pathogens which were not detected in their host blood samples [63]. Additionally, our study found *H. canis* DNA in ticks (*I. ricinus*), but not in the blood of the dogs, demonstrating that this pathogen circulates in Iasi, in the north-eastern part of Romania. The presence of co-infections with two or more TBPs, i.e., *Babesia* and *Hepatozoon*, may result in greater pathogenicity and more complications for the infected dogs. Despite of the availability of the ectoparasiticides and anti-feeding products, dogs are highly exposed to vector-borne diseases (VBD). There are many studies attesting that the majority of the dogs, not only the stray or kennelled dogs but owned dogs too, did not receive adequate preventative treatments in order to protect the animals from ticks, fleas, and sandfly or mosquito infestations [64,65,66]. Moreover, feline hepatozoonosis is an emerging VBD, and cats can be infected with *Hepatozoon felis* but also with *H. canis*. Thus, the protection of dogs could limit the circulation of pathogens (including *H. canis*) that could threaten the health of other animals [67].

In Romania, several studies reported different ranges of prevalence of *B. gibsoni* in symptomatic dogs from western and north-western Romania [31]. The lack of *B. gibsoni* infections in our study might be explained by a low number of cases of this infection in eastern Romania. Similar results were recently obtained in the southern part of Romania [10]. An unexpected outcome from the present study was the molecular identification of *B. rossi* in two symptomatic dogs. Indeed, one dog died and the other one was successfully treated. The main clinical signs displayed by the infected dogs were anorexia, fever, haemoglobinuria, anaemia, constipation alternating with diarrhoea, and splenomegaly. Actually, we cannot fully explain these results. However, the black-backed jackal (*Canis mesomelas*) is considered the natural reservoir host of *B. rossi* and *H. elliptica*, the most prevalent tick in jackal populations in the eastern and north-eastern parts of South Africa, and has the capacity of transmitting *B. rossi* [11]. A recent study revealed an unexpected outcome, showing no positivity of *B. rossi* in the black-backed jackal populations from South Africa [68]. To the authors’ knowledge, neither the vector or the pathogen of *B. rossi* was detected in Romania. However, the golden jackal (*Canis aureus*) is present throughout the country [69,70] and has already been incriminated as a potential natural reservoir of *Dirofilaria* spp. [71]. Perhaps, we can extend the hypothesis of the role of golden jackal populations or other wild canids such as wolves (*Canis lupus*) and red foxes (*Vulpes vulpes*) in maintaining the life cycle of *B. rossi* in Romania. In addition, the golden jackal is widespread in Europe, and an ongoing expansion of these wild canids has been reported in the Balkan Peninsula [72,73,74]. However, there are only three cases reported with *B. rossi* in Western Europe (France, Germany, and Switzerland) in a study that aimed to report the occurrence of *Babesia* spp. DNA in over 100,000 samples from America, Europe, Asia, and Oceania [75]. Further studies are needed to identify the tick species and their vector competence for canine babesiosis caused by *B. rossi* in Romania. The limitations of this study were as follows: (i) we collected mostly adult ticks from dogs, because this life-stage is easier to detect during the clinical examination. Molecular studies on larvae and nymphs could be useful to evaluate any differences in transmission of pathogens with adults; (ii) the small size of the blood samples collected from the symptomatic dogs and their ticks was not enough to identify the real picture of *H. canis* in the eastern part of Romania, knowing that there is high prevalence of *H. canis* infection in south, west, and central areas of Romania [8,10,18]; (iii) we identified only three *R. sanguineus* group ticks collected from dogs, which was an irrelevant number, in order to provide a conclusive screening on the actual spread of this tick in the studied area; (iv) we have not performed a follow-up of the two cases infected with *B. rossi* because one dog died and the owner did not approve the necropsy. The owner of the other dog that is still alive did not collaborate with us for further analyses; and (v) finally, we have not performed phylogenetic analyses.

## 4. Materials and Methods

### 4.1. Study Design and Samples Collection

The study was carried out in 2019 at the Clinics of the Faculty of Veterinary Medicine of Iasi, in the eastern part of Romania, on 90 owned dogs that showed clinical signs compatible with babesiosis (hyperthermia, haemolytic anaemia, thrombocytopenia, icterus, and haemoglobinuria) [21]. General information on the dogs’ age, breed, gender, tick infestation history, and outdoor access was provided by the owners. The ages of the dogs were grouped into five categories as follows: puppies (less than 1 year of age); young (1–3 years); adult (4–6 years); old (7–10 years); and very old (>10 years). All the dogs came from both urban and suburban areas of Iasi County and had no recorded history of travelling outside Romania.

EDTA-anticoagulated whole blood samples were collected from dogs that were enrolled and analysed for babesial parasites using quick Romanowsky stained thin blood smears. Briefly, thin air-dried blood smears were stained using a Diff Stain Quick kit (Pro-Eko S.r.l, Molise, Italy) and examined by an optical microscope at 1000X for detection of intra-erythrocytic piroplasms [23]. An aliquot of all the blood samples were stored at −20 °C until DNA was extracted for *Babesia* spp. and *H. canis* molecular detection.

Each dog was submitted to a clinical examination, and all detectable ticks were collected for species identification by standard taxonomic keys [76,77]. Moreover, life-stage, sex, and engorgement status of all the ticks collected from the coat of the dogs (No. = 15) were determined under a stereomicroscope. Additionally, 124 ticks collected from the coats of the other 23 dogs previously diagnosed with babesiosis by stained blood smears were included in the study. No information regarding the signalment data or clinical findings was provided for the dogs mentioned above. Moreover, the screening for *Babesia* species and *H. canis* in their blood was not performed. A scoring system has been used to express intensity of ticks per infested host, according to the World Association for the Advancement of Veterinary Parasitology (WAAVP) guidelines as follows (score-number of ticks/per animal) (None: 0; Low: 1–3; Moderate: 4–10; High: > 10) [78].

Collected ticks were stored in 70% ethanol and separated by individual animal for morphological detection.

All identified ticks (No. = 182) were divided into No. = 100 sub-samples (nine composed by pools of three ticks, 64 by pools of two ticks, and 27 sub-samples individually analysed) comprised of specimens collected from the same dog, and homogeneous species that were screened for *Babesia* species and *H. canis* using molecular analyses (Table 3).

### 4.2. DNA Extraction, PCR Amplification and Sequencing

All the blood and tick samples were transferred to the University of Federico II, Department of Veterinary Medicine and Animal Production, in Naples, Italy, where DNA extractions and molecular screenings were performed. DNA was extracted from 200 µL of blood in EDTA tubes (No. = 90 blood samples) and from ticks (No. = 100 pools) using the DNeasy Blood & Tissue kit (Qiagen, Leipzig, Germany) according to the manufacturer’s instructions. The ethyl alcohol was removed prior to DNA extraction. Quality and quantity of the extracted DNA were checked with a spectrophotometer (Multiskan GO, Thermo Fisher Scientific, MA, USA). The extracted DNA was stored at −20 °C. Specific primers were used to amplify the 18S rRNA gene fragment of *Babesia/Theileria* (559 bp): BabGF:5′-GYYTTGTAATTGGAATGATGG-3′; BabGR: 5′- CCAAAGACTTTGATTTCTCTC-3′ [79]; and *H. canis* (666bp): HepF:5′ ATACATGAGCAAAATCTCAAC -3′; Hep R: 5′-CTTATTATTCCATGCTGCAG -3′ [80].

Reactions were performed using the PCR protocol described by Bajer et al. [79] with some modifications for *Babesia* spp. Detection: a single end-point PCR was performed preparing a total 25 μL PCR volume (22 μL of PCR mix + 3 μL of the extracted DNA sample) for each sample with 1x buffer (EcoTaq PLUS, Lucigen, WI, USA) and 0.5 μM of each primer [79]. For *H. canis,* PCR was performed according to Inokuma et al. [80]. The PCR products were detected on a 1.5% ethidium bromide-stained low melting agarose gel (BIO-RAD, Spain). *Babesia canis* and *H. canis* DNA samples were used as positive controls, while PCR water was used as a negative control.

Since the tick sub-samples were composed of one–three ticks, the PCR results were expressed as a minimum infection rate (MIR), meaning the minimum percentage of ticks in a pool with detectable DNA for each specific pathogen. It was assumed that a PCR-positive sub-sample contains only one positive tick (also for sub-samples with two–three ticks) [81,82].

The amplified target DNAs for *Babesia* spp. and *H. canis* were purified with QIAquick PCR Purification Kit according to the manufacturer’s instruction (Qiagen, Hilden, Germany) and sequenced with forward and reverse primers (Eurofins, Martinsried, Germany). Sequencing results were analysed with the Chromas version 2.6.6 software (www.technelysium.com.au, accessed on 20 February 2019). DNA sequence comparisons were achieved by BLAST (www.ncbi.nlm.nih.gov, last accessed on 1 March 2021).

### 4.3. Statistical Analysis

Chi-square tests and confidence intervals at 95% (95% CI) were calculated using SPSS Statistics v.23 (IBM, Armonk, NY, USA) to verify the possible associations between dogs’ data (breed, age, gender, and access to outdoors) and the prevalence of the *Babesia* species. Differences were considered significant at *p* < 0.05.

## 5. Conclusions

The study revealed the first identification of *B. rossi* in two symptomatic dogs from Romania, although further studies are needed to confirm the presence of this pathogen and its vector in Europe and Romania and to investigate the vector competence of tick species that might be acting as vectors for this pathogen. Our study also demonstrated a high prevalence of *B. canis* and a low prevalence of *B. vogeli* in dogs and in their ticks in the eastern part of Romania; therefore, the genetic characterization of *Babesia* species could help practitioners select the appropriate testing and treatments, and for understanding the risks of infection, knowing that *B. canis* is considered more pathogenic than *B. vogeli*. Moreover, we can underline once more the importance of the molecular characterization of *Babesia* species and related pathogenicity in dogs, sustained as well by data that reveals the first description of a new piroplasmid species, *Babesia negevi* n. sp., in a dog with a fatal disease in southern Israel [27] and, recently, in Jordan too [83].

## Figures and Tables

**Table 1:** Gender, age categories, breed, lifestyle, clinical form of babesiosis, tick infestation history, exposure of ticks during the routine visit and prevalence of *Babesia* spp. for 90 dogs included in the study.

Variable	Babesial Prevalence (95% CI)
**Gender** Males Females	52.2% (41.5–62.7) 47.8% (37.2–58.5)
**Age categories (years)** Puppies (>1) 1–3 4–6 7–10 >10	5.6% (2.1–13.1) 58.9% (48.0–69.0) 13.3% (7.4–22.5) 10% (4.9–18.6) 12.2% (6.6–21.2)
**Dog breeds** Cross-breed Peking German Sheperd Akita Beagle Caucasian Sheperd Bullmastiff Bull terrier Boxer	52.2% (41.5–62.8) 14.4% (8.2–23.8) 10% (4.9–18.6) 2.2% (0.4–8.6) 5.6% (2.1–13.1) 4.4% (1.4–11.6) 1.1% (0.1–6.9) 3.3% (0.9–10.1) 6.7% (2.7–14.5)
**Lifestyle** Outdoors Indoors	74.4% (63.9–82.8) 25.6% (17.2–36.0)
**Tick infestation history**	100% (94.9–99.9)
**Dogs** With ticks Without ticks	16.7% (9.9–26.3) 83.3% (73.7–90.0)
**Babesiosis** Acute Mild	61.1% (50.2–71.0) 38.9% (28.9–49.8)
**Total dogs analysed (90)**	100% (94.9–99.9)

**Table 2:** Results of species and life-stages of ticks (total number collected and total pools) and pathogen species identified.

Species	No. of Ticks (Females/Males/Nymphs/Larvae)	No. Pools (Females/Males/Nymphs/Larvae)	Pathogen Species
*Ixodes ricinus*	112 (73/38/1/0)	64 (45/19/1/0)	* *B. canis* (*n* = 55) * *H. canis* (*n* = 15) * *B. microti*-like piroplasm (*n* = 1)
*Dermacentor reticulatus*	67 (39/28/0/0)	33 (20/13/0/0)	*B. canis* (*n* = 33)
*Rhipicephalus sanguineus* group	3 (1/0/1/1)	3 (1/0/1/1)	*B. vogeli* (*n* = 3)

* Co-infections: *B. canis + H. canis* (no. = 6); *H. canis + B. microti*-like piroplasm (*n* = 1).

**Table 3:** Study design.

Samples Collection	Laboratory Analyses
Blood samples from symptomatic dogs (no. = 90) and collection of ticks (no. = 58 ticks present on 15 dogs)	Quick Romanowsky stained-thin blood smear [23] for detection of intra-erythrocytic piroplasms DNA extraction, PCR amplification and sequencing [79,80] for *Babesia* spp. and *H. canis* Morphological identification of the ticks [76,77]
124 ticks collected from the coat of other 23 dogs previously diagnosed with babesiosis by stained blood smears	Morphological identification of the ticks [76,77]
Total number of tick sub-samples (no. = 100)	DNA extraction, PCR amplification and sequencing [79,80] for *Babesia* spp. and *H. canis*
